# Prevention and Control Strategies for Non-Communicable Disease: Goldberger, Pellagra and Rose Revisited

**DOI:** 10.3390/epidemiologia3020015

**Published:** 2022-04-06

**Authors:** John W. Frank

**Affiliations:** 1 Usher Institute, University of Edinburgh, Edinburgh EH8 9AG, UK; john.frank@ed.ac.uk; 2 Dalla Lana School of Public Health, University of Toronto, Toronto, ON M5T 3M7, Canada

**Keywords:** non-communicable disease, primary/secondary/tertiary prevention, primordial prevention, Geoffrey Rose, epidemiology, pellagra, Goldberger

## Abstract

This paper argues that the public health conceptual framework of epidemiologist Geoffrey Rose, first published as “Sick Individuals and Sick Populations” in 1985, provides a useful way to critically analyze prevention and control options for modern non-communicable diseases (NCD) and their forerunner, obesity, a pandemic now engulfing Lower-and-Middle-Income-Countries. That framework is based on the notions of primordial, primary, secondary and tertiary prevention—the full spectrum of “more upstream and more downstream” approaches, each with its pros and cons. These are illustrated using the pellagra epidemic in the southeastern USA from 1900 to the 1940s, which still has much to teach us about these same basic policy options for controlling the modern NCD pandemic. In particular, Rose’s dictum, “Seek the causes of (population) incidence, not of (individual) cases”, points up the compelling advantages of upstream prevention for controlling both epidemics.

## 1. Introduction

**Figure 1:**
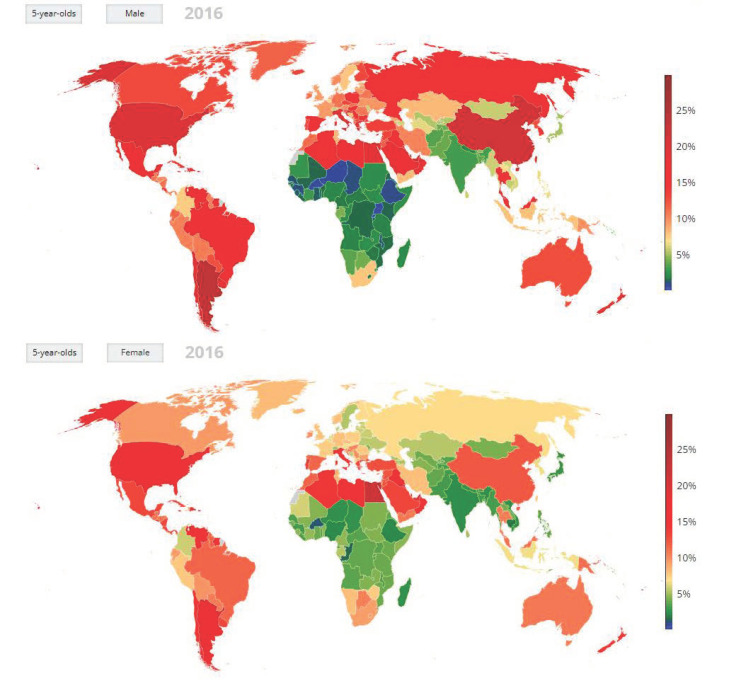
Global Prevalence Rates of Obesity in Five-Year-Olds (2016) [3].

Among Lower-and-Middle-Income Countries (LMICs), just decades after the “epidemiological transition” in High Income Countries (HICs) [1], non-communicable diseases (NCDs) are fast becoming the predominant cause of illness and premature death, presaged by a disturbingly rapid increase in child obesity [2,3] (Figure 1).

**Figure 2:**
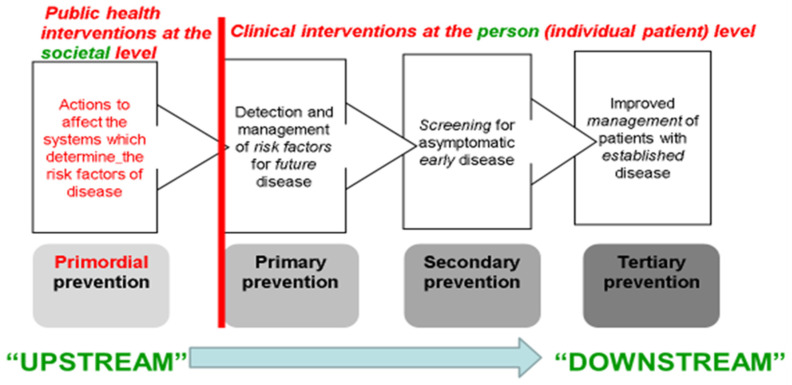
Four Types of Prevention Spanning Rose’s Two Strategies [7].

It is in this complex and still-evolving policy context that this essay considers the relative pros and cons of strategies for chronic disease prevention and control first enunciated by Geoffrey Rose, in his classic 1985 paper “Sick Individuals and Sick Populations” [4]. Rose, a British cardiovascular epidemiologist, founded the Whitehall Study of English civil servants—later analysed by his protégé, Sir Michael Marmot, to demonstrate persistent, protean and stepwise gradients in health across civil service pay-grades [5]. In Rose’s landmark 1985 paper, he lays out how chronic diseases can be tackled by two broad approaches that span “upstream”, population-wide prevention, involving public health interventions at the societal level, through to “downstream” clinical care focused on individuals at high risk—see Figure 2 [6].

## 2. Rose’s “Population Strategy”: Interventions at the Societal Level

***Primordial Prevention***, a term widely attributed to Strasser [7], was not part of Rose’s original formulation, but is easily integrated into it; it is defined as tackling the underlying reasons for the existence of chronic disease risk factors at the level of a whole society. For example, excessive salt consumption usually leads to widespread hypertension, a proven risk factor for cardiovascular disease [4,8]. Primordial prevention could therefore involve reducing salt in processed foods. Such population-wide interventions are normally the province of public health professionals, requiring concerted advocacy to achieve government and/or industry action to change food formulation and consumption patterns. This approach is also often termed “upstream” prevention [9].

## 3. Rose’s “High-Risk Strategy”: Interventions at the Individual Patient Level

The following three types of more clinically oriented prevention strategies—all of them constituting inherently more “downstream” actions—focus on individuals, usually via primary care. They were not all explicitly identified by Rose in his 1985 paper but have been extensively taught in epidemiology for decades [10].

***Primary Prevention*:** Identifying persons at high-risk, who have established risk factors for the chronic disease—e.g., hypertension as a risk factor for cardiovascular disease—and medically treating those risk factors (e.g., by weight loss, dietary change, exercise, and typically long-term pharmaceutical therapy, in the case of hypertension) to reduce those persons’ future risk of adverse disease outcomes. Public health professionals may be involved in such risk-factor screening and management programs at the community level, but much of this case-finding and management is done in routine primary care.

***Secondary Prevention*:** Identifying persons with early/asymptomatic disease, through screening programs, and treating them earlier in the disease’s natural history than would otherwise be the case, in the hope of improved outcomes (survival, quality of life)—sometimes these programs are managed by public health professionals, but often they also rely for delivery on primary care practitioners.

***Tertiary Prevention*:** Diagnosing and treating persons with the fully developed disease so as to prevent recurrences and complications—e.g., after the patient’s first episode of coronary heart disease or stroke, through prescribing long-term beta-blockers, anti-platelet drugs, statins, etc.; clearly this is a clinical approach, dependent on integrated secondary (hospital) and primary care systems, and ethically mandated as part of high-quality care for such patients.

We now examine how these contrasting approaches to chronic disease prevention and control can be instructively applied to a historical epidemic in the southeastern USA of a nutritional disease with a well-documented cause—pellagra (due to Vitamin B3 or nicotinic acid deficiency)—after which we will return to the current global NCD pandemic.

## 4. The Pellagra Epidemic in the US “Old South” (1906–1940)

**Figure 3:**
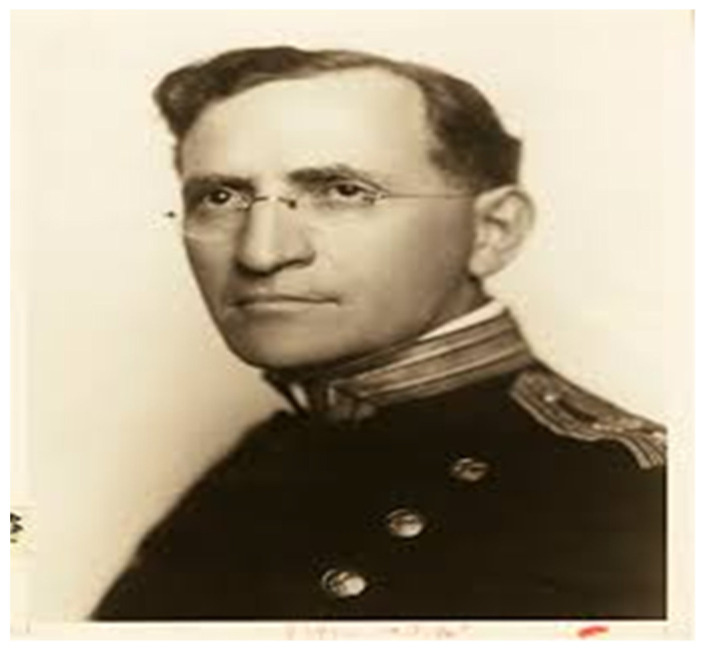
Dr. Joseph Goldberger (1874–1929). Source: Orthomolecular Hall of Fame: https://isom.ca/profile/joseph-goldberger/ (accessed on 31 March 2022).

In 1914, the US Surgeon General Rupert Blue asked US Public Health Service Officer Dr Joseph Goldberger (Figure 3) to formally take over the investigation and control of an epidemic of pellagra, which by that time had affected hundreds of thousands of victims in several Southern states, causing thousands of deaths, since its inception in 1906 [11]. Initially the outbreak had been covered up, since it first affected primarily inmates in institutions for the mentally ill, prisoners, and orphans. Because most victims were living in close proximity to each other, the dominant medical view at the time was that it was an infection. However, laboratory studies by a noted microbiologist, Dr Claude Lavinter, failed to disclose any of the usual characteristics of infectious disease. For example, the condition could not be transmitted in the laboratory to any experimental animal, nor even to human volunteers, by injecting or ingesting samples of tissues and excreta from human cases—standard “Koch’s Principles” for establishing an infectious agent as the cause of any disease [12]. 

Goldberger began his investigation by visiting the worst-affected prisons and asylums, as well as cotton-mill towns. He quickly saw that the common thread was not infection, as many believed, given two early observations:

(1) Even in the worst-affected institutions, there were no cases of the disease among employed staff—effectively ruling out infection as the cause.
(2) The main diet in all affected communities consisted of corn meal, biscuits, and molasses, sometimes served with meat, but usually only fatty portions of salt pork; fresh food was typically unavailable.

Within months, Goldberger’s further epidemiological investigations soon incriminated diet, in that: first, the institutional inmates were fed on the least expensive foodstuffs available; secondly, in cotton-mill “company towns”, workers were forced to purchase all their groceries from the company store, since they depended on credit until payday arrived, further limiting the sorts of foods they could afford. 

Goldberger, convinced that the disease was nutritional, then conducted several ingeniously designed experimental studies, to prove that hypothesis [13]. In 1914–15, he supplemented the diet of two orphanages and one part of an asylum for the mentally ill, where very high rates of pellagra had occurred. The following spring, when the annual peak of pellagra cases was expected, no new cases occurred in these institutions, and all the old cases had cleared. Unfortunately, when Goldberger’s funds ran out the following year, the disease returned, affecting 40% of children at the one orphanage—a kind of “clustered n-of-one” trial. However, Goldberger had made his point, fulfilling Bradford Hill’s most convincing criterion for causation: experimental reversibility [14]. 

The political context within which this investigation occurred speaks directly to the social determinants of health [5]. As word spread of Goldberger’s diagnosis of the epidemic as nutritional, powerful local vested interests began to resist the appalling notion that such a terrible outbreak could be due to inadequate diet, especially in workers employed in the economically important cotton industry [13]. Indeed, some of these notables pointed out that the disease was previously very rare in the southeastern USA, even though the diet implicated as its cause by Goldberger had been traditional for generations (an astute observation we will return to below, when we discuss primordial prevention of this epidemic). Influential local citizens could not accept that the disease was due to in large part to social conditions; it was far more palatable to believe it was caused by an infection [13]. 

Tragically, full acceptance of the dietary cause of pellagra did not occur until World War II. The disease gradually disappeared in the USA during the 1940s, as diets adequate in Vitamin B3 became the norm. The vitamin’s chemical identity was discovered in 1937, and within a few decades it became commonplace to fortify stable foods with it [13]. However, In the intervening two decades, between Goldberger’s definitive studies of prevention and the vitamin’s identification, hundreds of thousands of preventable pellagra cases and many deaths continued to occur. 

The twentieth-century outbreak of pellagra in the US demonstrates how the four hierarchical levels of prevention described above could have been implemented to control it. 

*Most ‘downstream’ disease control option* (*effectively tertiary prevention*)*:* Identify full-blown cases by regular house-to-house and institutional-resident surveys/examinations, and treat them all with an enriched diet: this approach would be the least expensive in the short run, since it focuses only on clinical cases rather than those at future risk—but it would do nothing to prevent further emerging cases in the rest of the population, who would continue to consume the same inadequate diet; therefore this intervention would have to be continued indefinitely into the future. As Rose pointed out [4], such a high-risk strategy is “palliative” not “radical”. 

*Slightly less* “*downstream*” *disease control option* (*effectively primary prevention*)*:* Focus on the communities and institutions with the highest rates of pellagra (thus improving efficiency and reducing programme costs), identifying individuals in those settings most at risk of pellagra, based on the risk markers Goldberger identified, such as age, gender, poverty, prior history, and inadequate diet; then enrich those persons’ diets, so as to prevent the emergence of new cases before they occur. This approach moves beyond treatment to prevention, but will not prevent all new cases, since some would inevitably emerge in settings not previously identified as high-risk. This strategy would also need to be continued indefinitely into the future, and so is likewise palliative. It also flies in the face of traditional dietary preferences—a feature of all high-risk strategies which, as Rose pointed out, is likely to reduce population compliance, as it attempts to change human behaviour which is effectively the cultural norm, but only in a subset of the population deemed at high risk, who may or may not appreciate their prescribed dietary change [4]. 

An even further “upstream” control option (a community-level version of primary prevention, but still not as far upstream as one could go to prevent future, similar outbreaks—see below): Consider all the poor residents of the counties or states affected by the outbreak to be at some risk of pellagra, and universally supplement all their citizens’ usual diets with milk, meat, legumes, and other natural sources of Vitamin B3. This option would be a more effective intervention overall, in that it would prevent virtually all new cases in the affected states’ populations. However, it would have been an expensive and logistically demanding approach, given that the Great Depression hit America shortly after scientific knowledge of the dietary cause of pellagra was first widely disseminated. While this strategy is radical for the high-risk communities whose diet is supplemented, it may not be acceptable to everyone, or logistically/economically feasible, since it requires dietary change amongst some persons at very low to no risk of pellagra within those populations. It is a hybrid “high-risk” and “population” strategy [4]. 

*The most ‘upstream’ option for preventive intervention:truly primordial prevention*: This fourth option can only come into play when there is clear scientific evidence of a much more fundamental driver of causation, addressing a very specific research question about the US 1906–1940 pellagra outbreak: *why did it began there and then*? As many proud Southerners pointed out to Goldberger, generations of poor locals had consumed an apparently identical diet: cornmeal, biscuits, fat-belly salt-pork, and molasses. What had changed just before the outbreak began in 1906? Bollet [13] points to strong circumstantial evidence that the culprit behind the American pellagra epidemic of that era was a *very specific technological change in food processing affecting corn meal, which occurred in the USA just after 1900:the development of a new corn-milling machine*. The Beall degerminator, patented in 1900–1901, was heralded as an improvement over traditional corn milling with grindstones, because it removed all the germ of the corn, thereby increasing transportability and shelf life, because the fatty content of the corn germ (corn oil) otherwise goes rancid over time. Unfortunately, many of the most nutritious components of corn, including Vitamin B3, are in the germ. In short, the US pellagra outbreak was probably precipitated, in borderline-malnourished sub-populations, by a manufacturing ‘advance’ that resulted in a mass-produced and nationally transported staple food at cheaper cost, but one severely deficient in a key nutrient. This explains why, as Goldberger perspicaciously observed, the cotton-mill towns with monopolistic company stores were much more affected by pellagra than nearby rural residents still milling their corn with traditional grindstones, which leave some of the germ in the corn meal.

Ironically, a similar epidemic, involving another dietary staple grain, rice, had taken place in the Far East a quarter-century earlier. In the 1880s, the advent of rice-milling machines, through European colonization, led to large outbreaks of another Vitamin B dietary-deficiency disease capable of killing its victims: beri-beri. Beri-beri is caused by thiamine (B1) deficiency. Rice milling removes the rice germ, the most nutritious part of the grain, but—as with corn—it is also the most perishable, because it is the oiliest. Remarkably, one scientist involved in the pellagra outbreak, Casimir Funk, did notice the similarity in the two outbreaks, and published his hypothesis before the conclusion of World War I, but was ignored [13].

## 5. Pellagra: A Personal Postscript

The author became clinically familiar with pellagra in the late 1970′s, while working as a Medical Officer at Mbeya Regional Hospital in southwest Tanzania. The disease presented initially precisely as in the USA: blackened, peeling skin on sites with sun exposure, followed by diarrhoea and, if untreated, dementia and death. Even a rapid search of the published literature reveals that pellagra has been documented in many parts of sub-Saharan Africa for over 70 years [15,16,17]. In the 1970s, we cured all the affected patients within days, with a simple injection of B vitamins costing only a few pennies. The author and his local medical colleagues were therefore pursuing mere *tertiary* prevention, doing nothing to fundamentally change the underlying drivers of the condition. As a result, it recurred at the end of each annual dry season, when the village larder was reduced to only long-stored corn, before the new harvest was ready. Indeed, the very patients we treated would typically present with the same symptoms again the following year, because water-soluble B vitamins cannot be stored in the body long-term.

Less well known is the fact that an even-further-upstream historical driver of pellagra had been set in place in Africa four centuries earlier. After Spanish and Portuguese traders brought corn (maize) to Africa from the New World, maize-based agriculture gradually forced out more traditional and nutritious staple crops (such as finger millet). Gross caloric yields per cultivated acre were much higher for corn, especially in areas with poor soils and little rainfall. Why did the adoption of corn as a staple in Africa lead to widespread pellagra, when that was not evident in its New World lands of origin, where native peoples had been eating corn as a staple for millennia? The answer is testimony to human cultural evolution, and the traditional wisdom of the many indigenous cultures who had survived on corn as for generations, apparently with no ill effects. It turns out that those ethnic groups who traditionally subsisted on corn in the Americas had a culinary trick, passed down through generations, which renders corn unlikely to cause pellagra if eaten in large quantities, as the staple food in an otherwise limited diet. Nutritional scholars eventually discovered that the treatment of corn meal with lime (obtained from burning limestone) converts an otherwise un-useable form of the amino acid tryptophan (a precursor of Vitamin B3) in corn meal into a form that can be used by the human body, greatly reducing the risk of pellagra [18]. However, when corn was brought to Africa—by non-agricultural and largely non-cooking sailors, slave-traders and merchants—no one thought to bring along with it the key culinary component for its preparation—lime. Clearly, the optimal primordial preventive intervention for pellagra in Africa would have been to teach everyone there to cook corn with lime, and/or find economically feasible ways to diversify the local diet with nutritious locally grown foods which are good sources of B vitamins, such as peanuts and beans. (Such massive dietary change is arguably much more difficult to achieve, in that societies which use corn as a staple love that food—cf. Rose’s point about shifting cultural norms [4].)

## 6. Conclusions

The four prevention and control strategies described above were specifically designed by Geoffrey Rose almost four decades ago [4] to tackle the then-emerging pandemic of non-communicable disease (NCD), now engulfing LMICs. Rather surprisingly, there is much to be learned about those strategies from studying perhaps the largest nutritional deficiency outbreak in an HIC of the entire twentieth century: pellagra in the southeastern USA, spanning the first four decades of that century. Were he still alive today, Rose might well simply reiterate his sage advice of 1985 to all public health professionals, to those now facing the current NCD pandemic: “Seek the (upstream) “causes of (population) incidence, not of (individual) cases” [4,8]. Doing that in the case of the NCD pandemic will require challenging conventional wisdom—in particular about the way food is currently grown, processed, marketed and consumed in industrialized economies [19,20,21,22,23]. Rose, and Goldberger before him, knew just how hard that would be.

## Data Availability

Not applicable.
